# The synergistic effect of the combination of polymyxin B and rifampicin in a murine neutropenic thigh infection model with *E. coli* and *K. pneumoniae*

**DOI:** 10.1093/jac/dkaf056

**Published:** 2025-02-26

**Authors:** Sanne Van Den Berg, Sebastiaan D T Sassen, William Couet, Sandrine Marchand, Heleen Van Der Spek, Marian T Ten Kate, Joseph Meletiadis, Anouk E Muller

**Affiliations:** Department of Medical Microbiology and Infectious Diseases, Erasmus MC, University Medical Center, Dr. Molewaterplein 40, 3015 GD Rotterdam, The Netherlands; CATOR, Center for Antimicrobial Treatment Optimization Rotterdam, Rotterdam, The Netherlands; CATOR, Center for Antimicrobial Treatment Optimization Rotterdam, Rotterdam, The Netherlands; Department of Hospital Pharmacy, Erasmus MC, University Medical Center, Rotterdam, The Netherlands; INSERM U1070, CHU de Poitiers et Université de Poitiers, Poitiers, France; INSERM U1070, CHU de Poitiers et Université de Poitiers, Poitiers, France; Department of Medical Microbiology and Infectious Diseases, Erasmus MC, University Medical Center, Dr. Molewaterplein 40, 3015 GD Rotterdam, The Netherlands; Department of Medical Microbiology and Infectious Diseases, Erasmus MC, University Medical Center, Dr. Molewaterplein 40, 3015 GD Rotterdam, The Netherlands; Department of Medical Microbiology and Infectious Diseases, Erasmus MC, University Medical Center, Dr. Molewaterplein 40, 3015 GD Rotterdam, The Netherlands; Attikon University Hospital, Medical School, National and Kapodistrian University of Athens, Clinical Microbiology Laboratory, Athens, Greece; Department of Medical Microbiology and Infectious Diseases, Erasmus MC, University Medical Center, Dr. Molewaterplein 40, 3015 GD Rotterdam, The Netherlands; CATOR, Center for Antimicrobial Treatment Optimization Rotterdam, Rotterdam, The Netherlands; Department of Medical Microbiology, Haaglanden MC, The Hague, The Netherlands

## Abstract

**Background:**

Antibiotic combination therapy is increasingly used to treat MDR pathogens. *In vitro* studies suggest that the polymyxin B/rifampicin combination might be synergistic. Therefore, the pharmacodynamics of rifampicin as monotherapy and combined with polymyxin B were studied in *Escherichia coli*- and *Klebsiella pneumoniae*-infected mice.

**Methods:**

The rifampicin pharmacokinetics (oral doses 0.5–64 mg/kg) in murine plasma were studied to estimate the exposures to rifampicin. These exposures were subsequently correlated with the antibacterial effect in a sigmoid maximum-effect model. The minimum exposures needed for a static, 1 log_10_ and 2 log_10_ kill effect in two *E. coli* and two *K. pneumoniae* strains were determined for monotherapy and the combination. The pharmacodynamic interactions between polymyxin B and rifampicin were assessed using Loewe additivity and Bliss independence in both an *E. coli* and a *K. pneumoniae* strain.

**Results:**

Rifampicin monotherapy resulted in a static effect in *E. coli* but not against *K. pneumoniae*. When combined with polymyxin B, rifampicin *f*AUC/MIC needed for stasis, 1 log_10_ and 2 log_10_ kill effect decreased with increasing polymyxin B exposures for all strains. Synergy was confirmed in Loewe additivity (interaction indices 0.11–0.51 for *E. coli* and 0.04–0.19 for *K. pneumoniae*) and Bliss independence (267% and 863%). Maximal killing (>2 log_10_ kill) in combination therapy was found at rifampicin/polymyxin B *f*AUC/MIC of 0.68/32.56 for *E. coli* and 0.169/16.28 for *K. pneumoniae.*

**Conclusions:**

These *in vivo* studies confirmed that there is a clear synergistic effect between polymyxin B and rifampicin, which was stronger for the *K. pneumoniae* strain than for the *E. coli* strain.

## Introduction

The rapid and ongoing spread of antimicrobial-resistant bacteria is considered a global threat. With the emergence of MDR pathogens and the limited number of novel antibiotics becoming available on the market, both carbapenems and polymyxins have been viewed as last-resort antibiotics.^1,2^ However, with the rise of strains harbouring MBLs, the effectiveness of the carbapenems is also reduced. To maximize the antibiotic effectiveness and to reduce the emergence of resistance, combination therapy has been recommended.^3^ Furthermore, combinations of old antibiotics may be an alternative to avoid expensive novel antibiotics. Since old antibiotics retain some *in vitro* activity against those pathogens, they were considered as alternative therapeutic options. However, for most of them, drug exposures are not sufficient to attain the preclinical pharmacodynamic targets.^4^ Combination therapy, if synergistic, may reduce drug exposures required for an antibacterial effect.

Polymyxin B is one of the polymyxin antibiotics. The relatively simple pharmacokinetics (PK) of polymyxin B, which is administered in its active form, is beneficial compared with that of colistin. Colistin is dosed as the inactive prodrug colistin methanesulfonate, which is slowly hydrolysed in the kidneys into active drug. It is active against Gram-negative pathogens, such as *Escherichia coli* and *Klebsiella pneumoniae* with MICs of <1 mg/L.^5,6^ However, pharmacodynamics (PD) studies of polymyxin B in a neutropenic murine infection model showed that the 1 log_10_ kill effect is reached only in a few strains.^7,8^ Polymyxin B use is limited due to toxicity issues with significantly higher nephrotoxicity at exposures (AUC_0–24_) above 100 mg·h/L.^9,10^ Efficacy of polymyxin could be increased and the risk of nephrotoxicity could be reduced with a combination therapy exerting synergistic effects where lower doses may be equally or more effective and less toxic than monotherapy. On the other hand, rifampicin monotherapy is not active against Enterobacterales, due to its inability to penetrate the bacterial outer membrane. Polymyxin B alters the outer membrane and this might also cause an increased ability for rifampicin to penetrate the outer membrane when administered in combination, explaining the synergistic effect between these two antibiotics.

The interaction between polymyxin B and rifampicin has been studied in several *in vitro* studies against Enterobacterles.^11,12^ Time–kill studies support the lack of activity of rifampicin and the limited effect of the polymyxins as monotherapy.^13,14^ Synergy has often been described in these models for the combination of rifampicin with polymyxin B or colistin.^12,15–17^ As in the clinical setting rifampicin is used in combination with other active agents against MDR isolates,^18^ it has been postulated that the combination between rifampicin and polymyxins is a promising combination to be used further in *in vivo* and clinical studies. Therefore, we determined the PD properties of polymyxin B, rifampicin and the combination in a neutropenic murine thigh infection model for *E. coli* and *K. pneumoniae*.

## Materials and methods

### Antibiotics and bacterial strains

Polymyxin B sulphate salt (lot 117M4045 V, 8926 units/mg; Sigma–Aldrich, Zwijndrecht, The Netherlands) was reconstituted in normal sterile saline (0.9% NaCl, Baxter, Utrecht, The Netherlands). Rifampicin (Rifadin, Sanofi, A7611) was reconstituted in water for injection. Both solutions were freshly prepared for each experiment. Two *E. coli* and three *K. pneumoniae* isolates with different resistance mechanisms were used (Table 1).

**Table 1.** Characteristics of the strains

Species	Strain	Resistance mechanisms	Median MIC (mg/L)	
			Polymyxin B	Rifampicin
*E. coli*	ATCC 25922	No	1	8
	15	CTX-M-15	0.5	16
*K. pneumoniae*	ATCC 43816	No	0.5	32
	17	SHV-1, OXA-1, CTX-M-15	1	32
	104	KPC-3, OmpK35red, OmpK36red	1	32

### *In vitro* susceptibility testing

The MICs of polymyxin B and rifampicin for each strain were determined in triplicate by broth microdilution, according to the CLSI guidelines.^19,20^ Median MIC values were reported and utilized in the PK/PD analysis.

### *In vivo* thigh and lung infection models in neutropenic mice

Experiments were carried out in accordance with the EU Animal Directive 2010/63/EU 2010^21^ (IRN 2019-0018), and approved by the institutional Animal Welfare Body, as previously described.^7^ Outbred female CD-1 mice (Charles River, Germany), with mean ± SD weight of 23.6 ± 1.6 g and aged 7–8 weeks, were used. Neutropenic mice (intraperitoneal cyclophosphamide injections of 150 and 100 mg/kg on Days −4 and −1, respectively), were infected intramuscularly in each thigh (thigh infection model) or intranasally under isoflurane anaesthesia (lung infection model), with a 0.05 mL inoculum (∼1 × 10^8^ cfu/mL).

Two hours after the infection (*t* = 0 h), treatment was started. All dosing regimens were performed in two animals per regimen. At 24 h after the first dose or when humane endpoints were reached (whichever came first), mice were humanely killed. Excised thighs or lungs were transferred to a pre-cooled 14 mL polypropylene tube containing 2 mL of PBS and were homogenized using a T25 ULTRA-TURRAX homogenizer (IKA-Werke GmbH & Co, Staufen, Germany). Ten-fold dilution series of the homogenate were prepared and 3 × 10 μL per dilution was plated on agar. After overnight incubation, colonies were counted and the number of cfu per thigh/lung was calculated.

### PK of rifampicin

To determine the PK of rifampicin, neutropenic thigh- or lung-infected mice were treated with single oral doses of rifampicin 2 h after infection (*t* = 0). Doses ranged from 0.5 to 64 mg/kg, two mice per dose. Four dose levels were studied in the thigh infection model (1, 4, 16, 64 mg/kg) and four dose levels were studied in the lung infection model (0.5, 2, 8, 32 mg/kg). Under isoflurane anaesthesia, blood was obtained at 12 different timepoints (one sample per mouse) in K3E EDTA tubes (Sarstedt, Nümbracht, Germany) through orbital sinus bleeding, which was immediately followed by cervical dislocation: at 0.083, 0.25, 0.5, 0.75, 1, 1.5, 2, 4, 6, 8, 10 and 12 h. Blood was centrifuged at 4000 rpm for 10 min and plasma was stored at −80°C until analysis.

### LC-MS/MS rifampicin assay

A previously described LC-MS/MS method was used for rifampicin assay in plasma and ultrafiltrate (UF) samples with slight adaptation.^22^ Plasma samples (50 µL) and 50/50 UF/plasma samples (50 µL) were precipitated by addition of 150 µL of acetonitrile containing rifampicin D8 (Alsachim, Illkirch Graffenstaden, France) at 50 ng/mL, used as internal standard. Transitions ions were *m*/*z* 823.5→791.0 for rifampicin and *m*/*z* 31.5→799.0 for rifampicin D8. The limit of quantification was 0.02 mg/L. The intraday variability was characterized at three concentrations with a precision lower than 9% and a bias lower than 14.5%. The corresponding between-day variability was characterized by a precision lower than 5% and a bias lower than 12%.

### Protein binding in murine plasma

Unbound concentrations of rifampicin were determined for a subset of the samples over the entire total concentration range of the experiment. The binding of rifampicin to plasma proteins was determined via ultrafiltration (4000 rpm for 30 min at room temperature) of 22 plasma samples containing rifampicin at total concentrations between 3.6 and 73.6 mg/L, using Centrifree^®^ ultrafiltration devices (Merck Millipore, Molsheim, France).

The total and unbound concentrations were plotted in Microsoft Excel (Microsoft Office Professional Plus 2019) and a trendline analysis for different equations was performed. The equation that best described the correlation between total and unbound concentrations was selected based on a visual inspection of the trendlines, as well as the R^2^ values. The correlation was described using the resulting formula, or the percentage of protein binding was calculated (mean ± SD).

### Pharmacokinetic modelling

All PK data were analysed simultaneously using non-linear mixed-effects modelling (NONMEM, version 7.4.4 ICON Development Solutions, Ellicott City, MD, USA) to develop a population PK model. The analysis was performed using the first-order conditional estimates method with interaction (FOCE + I). Prior to the analysis all total concentrations were converted to free concentrations and natural log-transformed. Data below the limit of quantification (LOQ) were omitted (<10%). Since dosages (in mg/kg) and mice were scaled to a virtual mouse of 1 kg, parameters were calculated for a virtual 1 kg mouse, resulting in PK parameters corresponding on a per kg base. The between-subject variability (BSV) on CL, *V* and absorption rate (*k*_a_) was described using an exponential model. Residual variability was described using a constant error model on natural log-transformed data. Changes in objective function values (dOFV), parameter precision, error estimates, shrinkages, condition number and visual inspection of the goodness-of-fit (GoF) plots and visual predictive checks (VPCs) were considered for model selection and validation. Model robustness was evaluated using bootstrap with resampling (*n* = 500) and fit to the data was evaluated using VPCs (*n* = 200).

Exposures in terms of AUC_0–24_ to be used in the PD analyses were determined by using the final population model. To translate the values to the unbound AUCs the formula for the protein binding as derived from the trendline analysis was included. Simulations (*n* = 1000) were performed for the different dosing regimen to determine the unbound AUCs (*f*AUC).

Exposures of polymyxin B in terms of *f*AUC to be used in the PD analyses were calculated based on previously published pharmacokinetic data in the same animal model using a protein binding of 80%.^7^

### PD of rifampicin and the combination polymyxin B/rifampicin

#### Exposure–response studies of rifampicin monotherapy

For exposure–response studies of rifampicin monotherapy, mice were infected with *E. coli* ATCC 25922, *E. coli* 15, *K. pneumoniae* ATCC 43816 or *K. pneumoniae* 17. Treatment regimens of 4–128 mg/kg total daily dose (TDD) given orally every 12 h, were used in the thigh infection model. The antibiotic effect was determined by comparing the log_10_ cfu/thigh values at *t *= 0 h (mean value of two mice) with the log_10_ cfu/thigh values at *t* = 24 h (mean value of two mice) and expressed as Δlog_10_ cfu/thigh. The Δlog_10_ cfu/thigh of rifampicin alone were plotted over the *f*AUC/MIC.

#### Exposure–response studies of combination therapy of polymyxin B/rifampicin

To study the combination of polymyxin B with rifampicin in the thigh infection model, mice were infected with *E. coli* ATCC 25922 and *K. pneumoniae* 104. Polymyxin B was dosed q6h over 24 h subcutaneously at three different dose levels (16, 32 and 64 mg/kg TDD), whereas rifampicin was dosed q12h orally at six different dose levels (1, 8, 16, 32, 64 and 128 mg/kg TDD). The chosen regimens in the combination therapy experiments were based on the dose–response curves of the rifampicin monotherapy as previously published for polymyxin B.^7^ Non-effective polymyxin B doses were combined with all rifampicin doses used in monotherapy. In the combination therapy experiments the highest doses of rifampicin (128 mg/kg TDD) and polymyxin B (64 mg/kg TDD) were used as monotherapy controls. The Δlog_10_ cfu/thigh of rifampicin in combination with polymyxin were plotted over the *f*AUC/MIC.

#### Pharmacodynamic drug interaction analysis

PD interactions were assessed based on Loewe additivity-based interaction index and Bliss independence-based response surface analysis. As AUC/MIC is the PK/PD index that best describes polymyxin B and rifampicin efficacy, pharmacodynamic analysis was performed using this index.^7,23^ A sigmoid maximum effect (E_max_) model was fitted to the *f*AUC/MIC versus Δlog_10_ cfu/thigh data by non-linear regression for all monotherapy and combination therapy regimens. As top, bottom and slope parameters did not differ significantly, those values were shared among the datasets and global fit was performed for the entire dataset of each isolate using GraphPad Prism 9.5.0 (GraphPad, Inc., San Diego, CA, USA). The static or a 1 log_10_ or 2 log_10_ kill effect were determined by interpolation for monotherapy and combination regimens.

##### Loewe additivity

An interaction index (I) was calculated for 2 log_10_ kill effects based on the Loewe additivity theory as I = EI_RIF + PMB_/EI_RIF _+ EI_PMB + RIF_/EI_PMB_, where EI_RIF_ and EI_PMB_ are the exposure indices *f*AUC/MICs of rifampicin and polymyxin B alone and EI_RIF + PMB_ and EI_PMB + RIF_ are the exposure indices *f*AUC/MICs of rifampicin in the presence of polymyxin B and of polymyxin B in the presence of rifampicin, respectively. As polymyxin B was inactive alone, the EI_PMB_ was determined from previous studies where a 350 and 977 *f*AUC/MIC was found to be associated with 2 log_10_ kill effects via extrapolation.^7^

##### Bliss independence

In order to capture interactions at the entire exposure–response surface, Bliss independence response surface analysis was used. The effect of rifampicin (E_RIF_) and polymyxin B (E_PMB_) alone and in combination (E_COMB_) were estimated as percentage of maximal log_10_ cfu/thigh reduction compared with drug-free controls. As polymyxin B was inactive (E_PMB_≈0%), the E_COMB_ was compared with the theoretical Bliss independent interaction E_IND _= E_RIF_ + E_PMB_ − E_RIF_ × E_PMB_ and for E_COMB _> E_IND_ or E_COMB _< E_IND_ synergy and antagonism, respectively, was concluded, otherwise Bliss independence was deemed. Statistical significance was assessed based on 95% CIs of E_MIN_, E_PMB_ and E_COMB_ determined by the E_max_ model. The 95% CI encompasses 95% of future datapoints if the isolate will be tested 100 times.

## Results

### Plasma protein binding of rifampicin

The protein binding was determined in a subset of the samples with concentrations from 3.6 to 73.63 mg/L. Overall, the murine plasma protein binding was >99%. The correlation between the total and unbound plasma concentrations was best described by a power model (R^2^ = 0.7281), resulting in the following formula: unbound concentration = 0.0166 × (total concentration)^0.8162^. One datapoint was excluded from the analysis (extreme outlier, total concentration was >3 SD from the mean).

### PK of rifampicin

**Figure 1.**
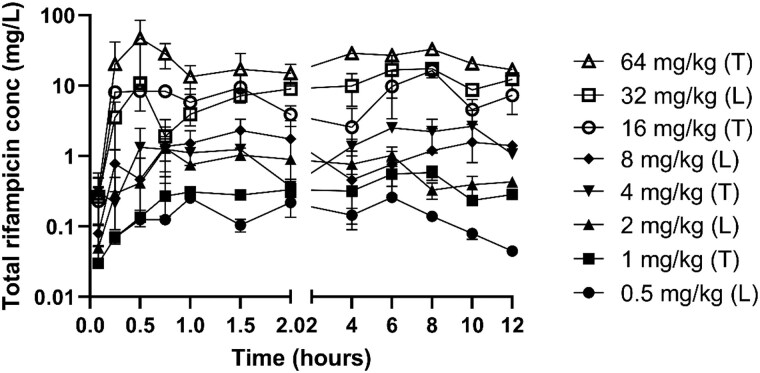
Single-dose plasma PK of oral rifampicin in neutropenic murine thigh (T) and lung (L) infection models.

The PK profile of rifampicin total concentrations in mouse plasma after single oral administrations of 0.5–64 mg/kg is shown in Figure 1. The clearance is low, resulting in levels above the detection limit during the entire experiment (12 h) for all dosing regimens. A total of 219 murine plasma samples were included in the analysis, of which 3 were excluded (<LOQ). The PK analysis was performed using the unbound concentrations. The best PK model was a one-compartment model with a first-order elimination and absorption with BSV on *V*. BSV on CL, *k*_a_ or combinations including *V* did not improve the model. Residual error was described with a constant error (natural log scale). The shrinkages for the residual error were high, which is expected considering that only a single datapoint per mouse can be obtained.

The final model was validated using bootstrap and VPC and showed an overall reasonable fit (Figure S1, available as Supplementary data at *JAC* Online). The overall VPC shows a slight overestimation of variability and observations showed a pulldown at around 4 h after the dose. The result of the bootstrap was in line with the final model. The final parameter estimates and the results of the bootstrap are shown in Table 2.

**Table 2.** Estimates of the final PK model of total concentrations and the results of the bootstrap

	Final model			Bootstrap	
Parameter	estimate (SE)	RSE (%)	Shrinkage	estimate (SE)	95% CI
*V*_c_/F (L/Kg)	169 (36.8)	21.8	—	169 (29.5)	109–208
CL/F (L/kg/h)	7.4 (2.7)	36.8	—	7.2 (2.1)	4.3–11.5
*k*_a_ (h^−1^)	1.11 (0.47)	42.3	—	1.14 (0.41)	0.51–1.92
BSV *V* (%)	89.4	11.9	14	90.9	68.8–115.6
Residual error	0.33 (0.13)	24.0	49	0.30 (0.07)	0.02–0.82

*V*_c_/F, central apparent *V*; CL/F, apparent clearance; *k*_a_, absorption rate constant; residual err, constant residual error; SE, standard error; RSE, relative standard error.

### PD of rifampicin

**Figure 2.**
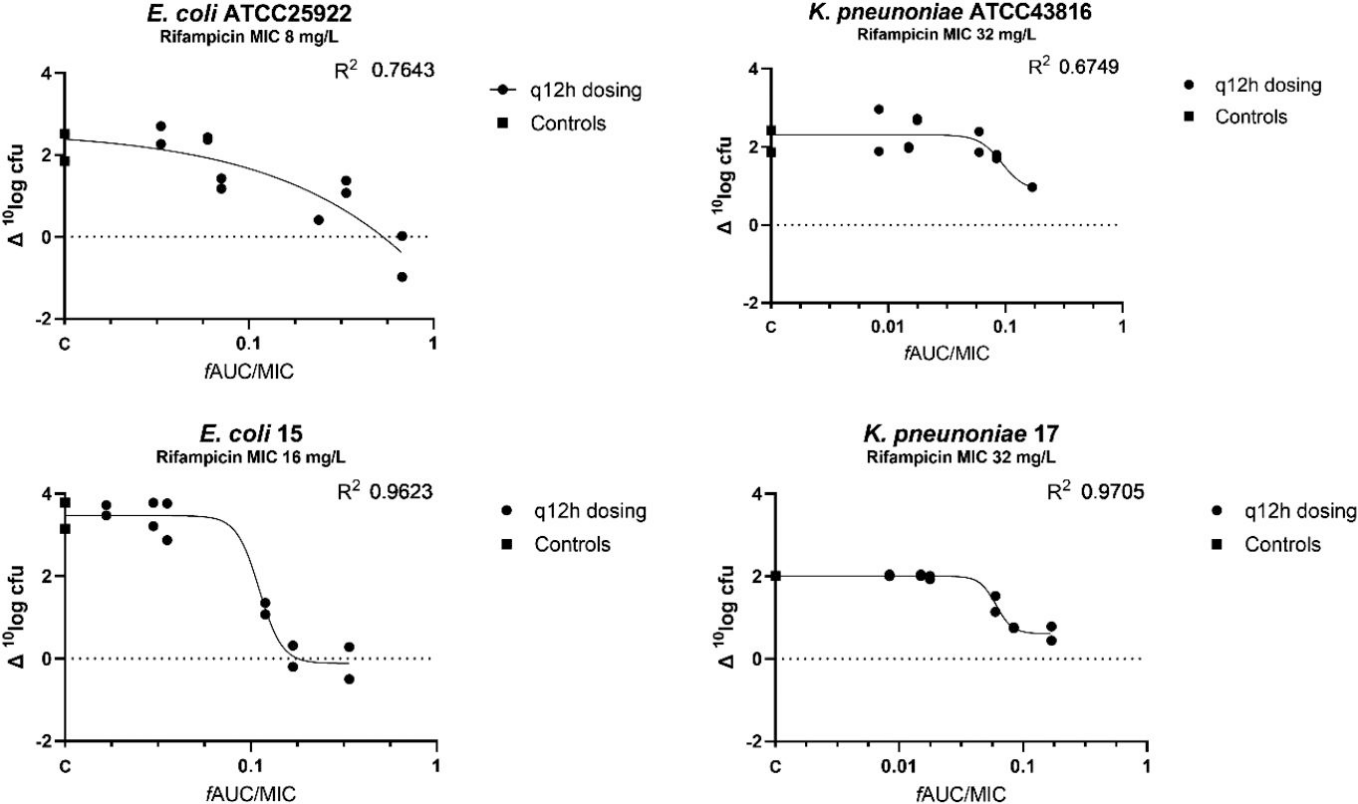
Change in cfu versus plasma *f*AUC/MIC ratio for four Enterobacterales strains in the thigh infection model. The change in bacterial load in an individual thigh is represented by a datapoint. Controls (C) represent the placebo-treated controls.

The exposure–response curves in mice treated with rifampicin against two *E. coli* and two *K. pneumoniae* strains are shown in Figure 2. The average bacterial load at the start of treatment was 1.9 × 10^7^ (range 1.0–3.2 × 10^7^) cfu/thigh. For the two *E. coli* strains, a static effect was obtained, whereas this was not reached in the two *K. pneumoniae* strains. The 1 log_10_ kill target was achieved in one of the four strains. The exposure–response relationships were well described by the E_max_ model (R^2^ = 0.675–0.971) and 1 log_10_ growth, static and 1 log_10_ kill targets were calculated. The resulting *f*AUC/MIC ratios correlating with 1 log_10_ growth, stasis and 1 log_10_ kill are shown in Table 3. When all strains were co-modelled, the *f*AUC/MIC correlated with 1 log_10_ growth was 0.11. For the two *E. coli* strains, *f*AUC/MIC correlating with stasis was 0.42. Due to the fact that monotherapy with rifampicin had very limited activity on Enterobacterales, 58% of the rifampicin-treated mice were terminated before *t* = 24 h as humane endpoints were reached.

**Table 3.** Rifampicin monotherapy *f*AUC/MIC ratios and effect of the different strains

Strains	1 log_10_ growth	stasis	1 log_10_ kill
*E. coli* ATCC 25922	0.24	0.53	0.93
*E. coli* 15	0.12	0.18	NR
*K. pneumoniae* ATCC 43816	0.16	NR	NR
*K. pneumoniae* 17	0.07	NR	NR
Co-modelled data:			
* *Two *E. coli* strains	0.11	0.42	NR
* *Two *K. pneumoniae* strains	0.13	NR	NR
* *All four strains	0.11	NR	NR

NR, not reached.

### PD of polymyxin B/rifampicin combination

**Figure 3.**
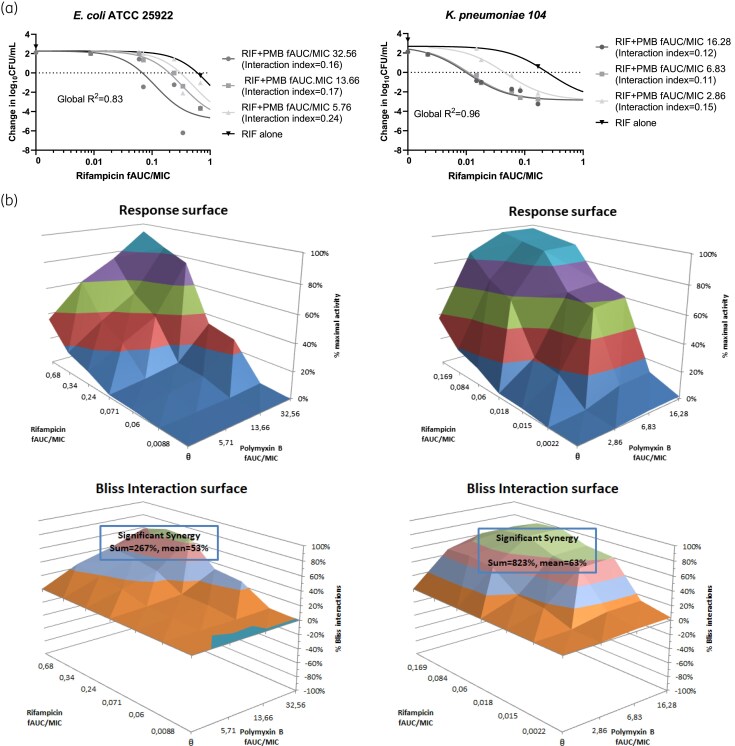
Exposure–response relationships of rifampicin and polymyxin B alone and in combination. (a) Loewe additivity; (b) Bliss interaction surface.

In the combination experiments, where mice were treated with polymyxin B in combination with rifampicin, an effect of increasing doses of polymyxin B on rifampicin exposure–response curves has been detected, with a shift of the curves to lower exposures as polymyxin B exposure increased. When rifampicin was combined with 8 or 16 mg/kg polymyxin B, 2 log_10_ kill was reached against both the *E. coli* and the *K. pneumoniae* strains, and when rifampicin was combined with 4 mg/kg polymyxin B, 1 log_10_ kill was reached against the *E. coli* strain and 2 log_10_ kill against the *K. pneumoniae* strain. The exposure–response curves for the combination are shown in Figure 3. The *f*AUC/MIC needed for a static, 1 log_10_ kill and a 2 log_10_ kill target effect decreased with an increasing exposure of polymyxin B (Table 4). Polymyxin B was inactive alone (no cfu reduction) whereas rifampicin alone produced a static effect. The interaction indices for 2 log_10_ kill effects ranged from 0.16 to 0.24 for *E.coli* and from 0.11 to 0.15 for *K. pneumoniae*, indicating synergistic effects, which were stronger against *K. pneumoniae*. This was also verified with Bliss independence analysis, where the sum of statistically significant interactions was 267% for *E. coli* (mean 53%) and 823% for *K. pneumoniae* (mean 63%). The maximal killing found for combination therapy was −4.89 at rifampicin/polymyxin B *f*AUC/MIC 0.68/32.56 against *E. coli* and −2.78 at rifampicin/polymyxin B *f*AUC/MIC 0.169/16.28 against *K. pneumoniae*, when monotherapy regimens hardly reached stasis for rifampicin whereas no effect was found for polymyxin B alone.

**Table 4.** Rifampicin combination therapy *f*AUC/MIC ratios for different levels of effect for two strains and three dose levels of polymyxin B

Strain	Polymyxin B *f*AUC/MIC^a^	Rifampicin *f*AUC/MIC target for		
		Stasis	1 log_10_ kill	2 log_10_ kill
*E. coli* ATCC 25922	0	0.606	0.895^b^	1.297^b^
	5.71	0.308	0.456	0.660
	13.66	0.198	0.293	0.424
	32.56	0.068	0.101	0.146
*K. pneumoniae* 104	0	0.240^b^	0.428^b^	0.954^b^
	2.86	0.045	0.081	0.180
	6.83	0.011	0.020	0.044
	16.28	0.010	0.018	0.040

^a^Data from Van der Meijden *et al*.^7^ The exposure indices associated with stasis, 1 and 2 log_10_ kill was extrapolated to be 95, 180 and 350 *f*AUC/MIC for *E. coli* and 16, 83 and 977 *f*AUC/MIC for *K. pneumoniae*.

^b^Rifampicin alone resulted in stasis at the highest dose tested. The exposure indices associated with 1 and 2 log_10_ kill effects were extrapolated using the Emax model.

## Discussion

In a murine thigh infection model, rifampicin monotherapy was shown to have a limited effect on *E. coli* and *K. pneumoniae*. With both *E. coli* strains, a static effect was reached, where this was not reached for the *K. pneumoniae* strains. From our previous study it is known that static effect can be expected for polymyxin B monotherapy for *E. coli* and *K. pneumoniae*, and for a minority of the strains 1 log_10_ kill was reached.^7,8^ The combination was clearly synergistic due to the increase in potency (lower effective exposures) and efficacy (increased killing) of antibacterial effects when rifampicin and polymyxin B were combined, as confirmed with the Loewe additivity and Bliss independence analysis.

The *f*AUC/MIC values used in the monotherapy experiments for rifampicin appear to be low compared with previous studies. However, this can largely be explained by the high protein binding of >99% found in the current study. The PK analysis was performed based on unbound concentrations, and since different percentages of protein binding were also reported in the literature^14^ and data are often presented as total values, it is difficult to compare. The murine PK studies were performed up to 12 h after the dose administration. As the concentrations at the end of the experiment were still relatively high, accumulation was expected. To account for the accumulation, simulations were performed using the population model to determine the exposure 0–24 h. Overall the *f*AUC values from the current study compared well with a previous study in ICR mice.^24^ Another study used *K. pneumoniae* strains (rifampicin MICs of 32–128 mg/L), administered as a single intraperitoneal injection of 25 mg/kg rifampicin. They found no effect in the peritoneal sepsis model, but this dose was found to be effective in a pneumonia model for a strain with an MIC of 32 mg/L.^14^ In that study, plasma AUC was determined in healthy mice and found to be 34.5 mg·h/L. This translates to an *f*AUC/MIC of 1.38 mg·h/L, given the average protein binding of 96% used in their study.^14^ This *f*AUC/MIC after intraperitoneal injection is higher compared with values we used in the analysis after oral rifampicin administration. The previous study used only one dose level and therefore did not determine the minimal AUC/MIC required for efficacy and they did not show data in the thigh model, which we used in the present study. These differences may explain the differences between our study and the previous study.

To compare with our results, limited data are available on the combinations from *in vivo* studies and their results are conflicting. No added value was demonstrated in a *Galleria mellonella* model of infection when polymyxin B was combined with rifampicin over polymyxin B monotherapy in the treatment of KPC-producing *K. pneumoniae*.^25^ In a mouse model of *Pseudomonas aeruginosa* pneumonia, a synergistic effect between colistin and rifampicin was shown in the survival rates; however, this effect was limited to the intranasal administration of colistin. There was a discrepancy in the survival rates between the groups of mice receiving colistin intranasally (survival rate 100%) and subcutaneously (survival rate 14%; significantly different, with *P* < 0.01).^26^ Both colistin regimens were combined with oral rifampicin. This discrepancy in survival rates is likely caused by differences in exposure at the site of infection due to PK characteristics of colistin. The finding is in line with the results of Levin *et al*.,^27^ who showed that IV colistin was less effective against pneumonia (25% cure) than other sites of infection (75% cure) in the treatment of *P. aeruginosa* and *Acinetobacter baumannii*. Of note, in clinical practice many MDR isolates might also harbour resistance to the carbapenems, but this is unlikely to influence the potential synergistic interaction between polymyxin B and rifampicin.

A clinical trial in eight patients with pneumonia caused by colistin-resistant *A. baumannii* compared colistin monotherapy with the colistin/rifampicin combination.^28^ Colistin resistance was determined using a gradient test, which is known to be unreliable for colistin.^29^ The microbiological response in the combination group was 100%, whereas this was 40% in the colistin monotherapy patients. Due to the limited number of patients in the study, the difference in microbiological response was not statistically significant.^28^

Among the limitations of the current study is the fact that the pharmacokinetic population model indicated that there was most likely a saturable absorption for the highest dose level. Therefore, the simulated values for the AUC for this dose level might be overestimated. However, this will not influence the target values for stasis, 1 log_10_ kill and 2 log_10_ kill. Another limitation is that the combination study was performed using two strains, since the number of strains that can be used in animal experiments is limited for ethical reasons. The results of both strains were in line with each other showing a synergistic effect for the combination.

In a review on the PK of rifampicin in tuberculosis patients, the mean total AUC at steady state after a 10 mg/kg dose was found to be 38.73 mg·h/L.^30^ The human protein binding was reported to be 70%–90%.^30–32^ Taking into account a protein binding of 80% and an MIC of 32 mg/L, the mean *f*AUC/MIC for this dose is 0.242, which exceeds the rifampicin *f*AUC/MIC of 0.047–0.136 needed for 2 log_10_ kill in the current study when combined with the high dose polymyxin B that corresponds to an *f*AUC/MIC of 16.28–32.56. Based on the exposures attained with the standard dose of 50 mg q12h of polymyxin B (AUC = 75 mg·h/L, unbound fraction = 0.42),^33^ the latter PK/PD target of polymyxin B can be attained for *E. coli* and *K. pneumoniae* isolates with MICs up to 1–2 mg/L, which encompass the MIC_90_ values for both pathogens (0.5–1 mg/L).^6,34^

This study showed limited activity of rifampicin and polymyxin B monotherapy against *E. coli* and *K. pneumoniae* and supported the synergistic effect of the combination polymyxin B/rifampicin in a murine infection model at clinically achievable exposures. Currently, dosing of polymyxin B is limited by nephrotoxicity.^35^ Therefore, optimizing the polymyxin B dose and avoiding nephrotoxicity needs to be balanced and might be challenging. The current finding might indicate that due to the synergistic effect of the combination of polymyxin B with rifampicin, lower doses of polymyxin are required, and therefore the achievement of optimal (combination) target values can be reached with a reduced risk of nephrotoxicity. Future studies are required to test whether this synergistic effect is observed in other Enterobacterales and whether this effect can be seen in the majority of the isolates.

## Supplementary Material

dkaf056_Supplementary_Data

## Supplementary data

Figure S1 is available as Supplementary data at *JAC* Online.
