# Supplementary material for: The synergistic effect of the combination of polymyxin B and rifampicin in a murine neutropenic thigh infection model with E. coli and K. pneumoniae
Source: J Antimicrob Chemother. 2025 Feb 26;80(5):1248–55. doi: 10.1093/jac/dkaf056 (PMC12046403; doi:10.1093/jac/dkaf056)
Supplement: dkaf056_Supplementary_Data [file dkaf056_supplementary_data.docx]

Figure S1: VPC of the final model


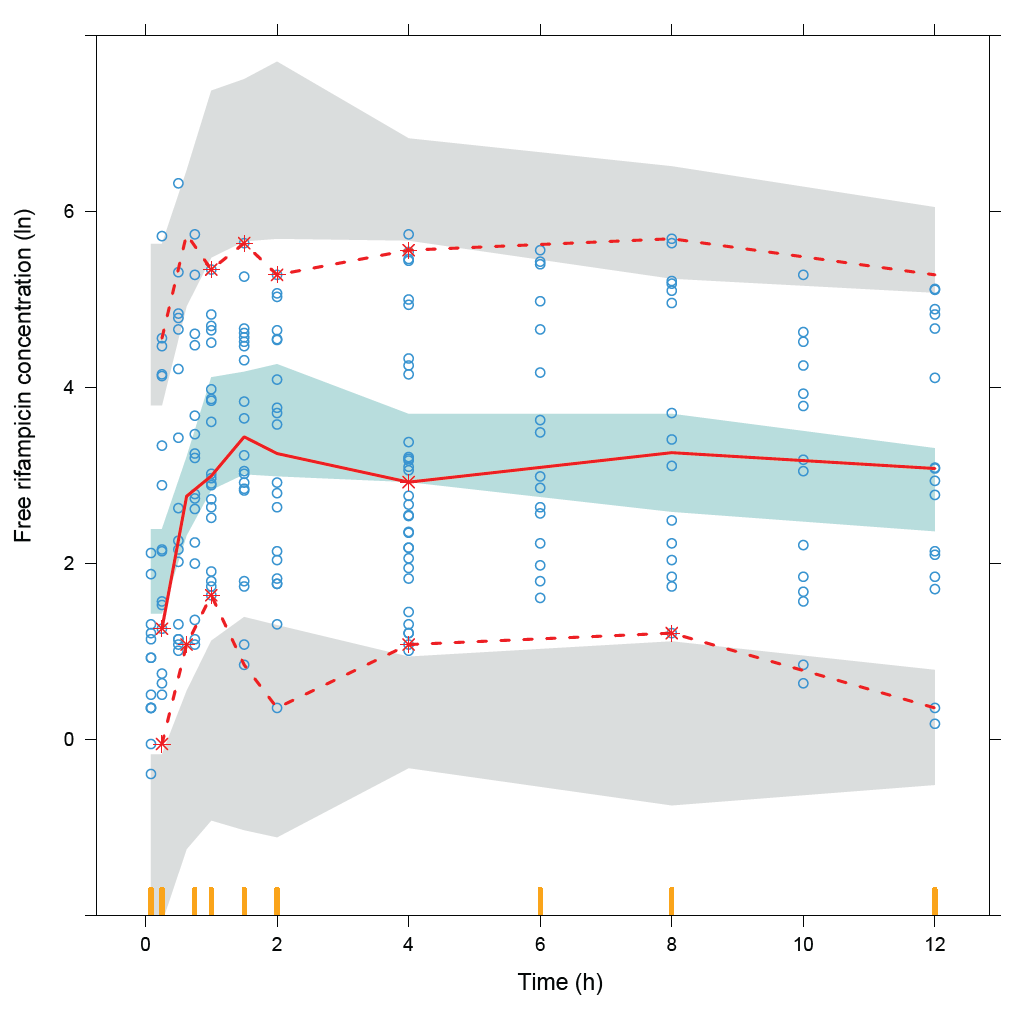


Figure S1

Simulations: Grey areas: 5^th^ and 95^th^ percentiles; blue area: median; Observations: solid line: median. Dotted lines: 5^th^ and 95^th^ percentiles. The yellow bars indicate the binning used in the analysis.
